# MFPPDB: a comprehensive multi-functional plant peptide database

**DOI:** 10.3389/fpls.2023.1224394

**Published:** 2023-10-16

**Authors:** Yaozu Yang, Hongwei Wu, Yu Gao, Wei Tong, Ke Li

**Affiliations:** ^1^ School of Information and Computer, Anhui Agricultural University, Hefei, China; ^2^ Information Materials and Intelligent Sensing Laboratory of Anhui Province, Anhui University, Hefei, Anhui, China; ^3^ State Key Laboratory of Tea Plant Biology and Utilization, Anhui Agricultural University, Hefei, Anhui, China; ^4^ Anhui Provincial Engineering Laboratory for Beidou Precision Agriculture Information, Anhui Agricultural University, Hefei, Anhui, China

**Keywords:** multi-functional, therapeutic peptide, plant, database, peptidomes

## Abstract

Plants produce a wide range of bioactive peptides as part of their innate defense mechanisms. With the explosive growth of plant-derived peptides, verifying the therapeutic function using traditional experimental methods are resources and time consuming. Therefore, it is necessary to predict the therapeutic function of plant-derived peptides more effectively and accurately with reduced waste of resources and thus expedite the development of plant peptides. We herein developed a repository of plant peptides predicted to have multiple therapeutic functions, named as MFPPDB (multi-functional plant peptide database). MFPPDB including 1,482,409 single or multiple functional plant origin therapeutic peptides derived from 121 fundamental plant species. The functional categories of these therapeutic peptides include 41 different features such as anti-bacterial, anti-fungal, anti-HIV, anti-viral, and anti-cancer. The detailed physicochemical information of these peptides was presented in functional search and physicochemical property search module, which can help users easily access the peptide information by the plant peptide species, ID, and functions, or by their peptide ID, isoelectric point, peptide sequence, and molecular weight through web-friendly interface. We further matched the predicted peptides to nine state-of-the-art curated functional peptide databases and found that at least 293,408 of the peptides possess functional potentials. Overall, MFPPDB integrated a massive number of plant peptides have single or multiple therapeutic functions, which will facilitate the comprehensive research in plant peptidomics. MFPPDB can be freely accessed through http://124.223.195.214:9188/mfppdb/index.

## 1 Introduction

Since the introduction of insulin therapy in the last century, peptide therapies have also been used in medical treatment in recent years, and dozens of peptide drugs have been approved to enter the market (Muttenthaler et al., 2021). For example, the peptides for oral use (Kong et al., 2020), a long-acting glucagon-like peptide-1 in healthy male subjects (Elbrønd et al., 2002), an anti-cancer peptide targeting the transcription factor FOXM1 (Cheng et al., 2023), the cyclic peptide drugs (Zhang and Chen, 2021), peptide Etelcalcetide indicated for secondary hyperparathyroidism (Hamano et al., 2017). Therapeutic peptides have been used to treat various diseases such as cancer, diabetes, HIV, and cardiovascular diseases (Wang et al., 2022). Historically, therapeutic peptides were defined as peptides with 2 to 50 amino acids, which play a essential therapeutic role in the human body (Henninot et al., 2018). However, it was also noted that peptide length might not be a serious limitation for peptide drug development (Lau and Dunn, 2018).

Plant proteins contain an average of 350 to 400 amino acids (Ramírez-Sánchez et al., 2016), while longer peptides are universally toxic and less stable (Kim et al., 2014). Plants possess many kinds of defense mechanisms to combat the physical, chemical, and biological coercion they face in nature (Das et al., 2020). Plant-derived bioactive peptides have potentially wide applicability in the agrochemical and pharmaceutical industries and are a new and unexplored area in the field of proteomics and peptidomics (Sarethy, 2017). An enormous amount of therapeutic peptides with potential functions for the treatment of human diseases have been identified from plant sources, making plant peptides may become a new beginnings for therapeutic peptides (Chai et al., 2021).

In order to facilitate the comprehensive application of therapeutic peptides, various databases of therapeutic peptides have been established over the decades, such as APD3 that includes the antimicrobial peptides (Wang et al., 2016); PhytAMP, dedicated to plant antimicrobial peptides (Hammami et al., 2009); PlantPepDB, which include the manually curated plant peptides (Das et al., 2020); ACovPepDB, which collected anti-coronavirus peptides (Zhang et al., 2022a); PlantAFP, which collected curated plant-origin antifungal peptides (Tyagi et al., 2019); CancerPPD, a database of anticancer peptides and proteins (Tyagi et al., 2015); AHTPDB for analysis and presentation of antihypertensive peptides (Kumar et al., 2015); BIOPEP, database of sensory peptides and amino acids (Iwaniak et al., 2016); DAMPD, a manually curated antimicrobial peptide database (Seshadri Sundararajan et al., 2012); THPdb, which collectes FDA-approved peptide and protein therapeutics (Usmani et al., 2017); and YADAMP, another database of antimicrobial peptides (Piotto et al., 2012). While, PlantPepDB, PhytAMP, and PlantAFP are all databases of plant-derived therapeutic peptides; PhytAMP only contains antimicrobial peptides; PlantAFP just contains plant-derived antifungal peptides. Although PlantPepDB is a plant-specific database with a variety of therapeutic and bioactive functions, it currently only includes 3,848 peptides, of which only 1,465 are therapeutic peptides.

Identifying the therapeutic functions of plant peptides through traditional experimental methods can be both expensive and time-consuming. To address this issue, we propose the Multi-Functional Plant Peptides Database (MFPPDB), a database of plant peptides predicted to contain multiple therapeutic functions. With the MFPPDB, researchers can more efficiently identify plant peptides with potential therapeutic functions, reducing the scope and cost of identification. MFPPDB provides comprehensive information on multifunctional plant peptides, including information on their functional characteristics, detailed physicochemical properties, and a universal multifunctional prediction server. It contains 1,482,409 plant peptides with single or multiple therapeutic functions derived from represented plant databases, i.e. TPIA (Xia et al., 2019), TAIR (Lamesch et al., 2012), RGAP (Kawahara et al., 2013), SGN (Zhou et al., 2022), SpudDB (Pham et al., 2020), IWGSC (Consortium et al., 2014) and Phytozome (Goodstein et al., 2012). The establishment of MFPPDB will not only facilitate the identification of multifunctional plant peptides, but also accelerate the research and development of therapeutic peptide drugs, making them more accessible to the market. Furthermore, it will also attract researchers from a wide range of fields involved in the research and development of peptide drugs.

## 2 Results and discussions

### 2.1 Functional peptide characterization

**Figure 1:**
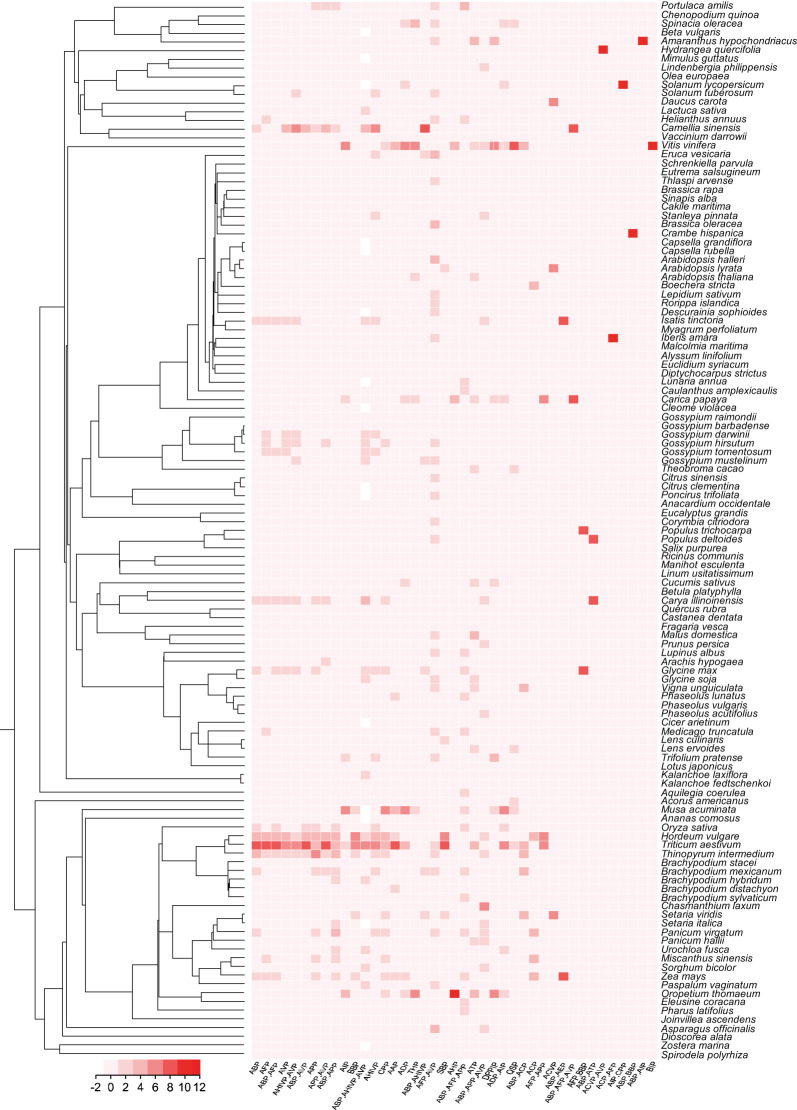
The phylogenetic relationship and the number of functional therapeutic peptides categories of plant species in MFPPDB database. The heatmap indicates the number of functional peptides contained in each species.

**Figure 2:**
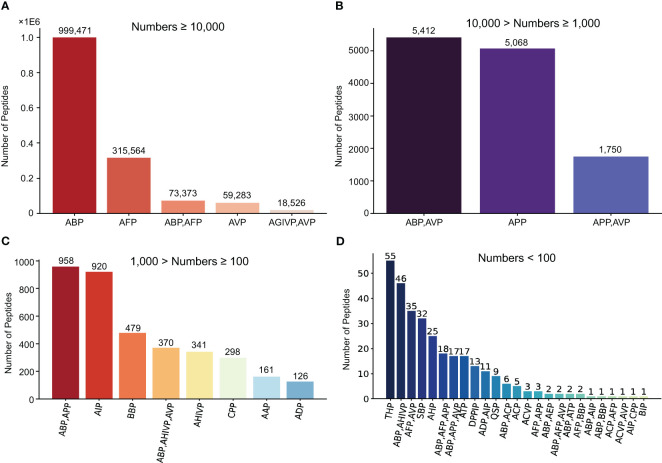
All the functional peptide types and corresponding quantities contained in MFPPDB database. We classified the peptide types into four group according to the quantity. **(A)** The five functional peptides with quantity more than 10,000, **(B)** The three functional peptides with quantity between 1000 and 10,000, **(C)** The eight functional peptides with quantity between 100 and 1000, **(D)** 25 functional peptides with quantity below 100.

The MFPPDB database applied a modifying PrMFTP (Multi-functional therapeutic peptides) algorithm based on multi-head self-attention mechanism and class weight optimization to identify the peptides from 121 plant species (Yan et al., 2022). A total of 1,482,409 peptide sequences with therapeutic functions were finally obtained, with average 12,251 in each species (**Figure 1**). The therapeutic function category includes 21 unique functional characteristics, such as anti-angiogenic peptide (AAP) (Zhang and Zou, 2020), anti-bacterial peptide (ABP) (Xiao et al., 2021), anti-cancer peptide (ACP) (Agrawal et al., 2021), anti-coronavirus peptide (ACVP) (Pang et al., 2021), and anti-diabetic peptide (ADP) (Roy and Teron, 2019). These peptides were classified into 41 therapeutic peptides categories with different functional characteristics (**Table 1**). Each species has one or more peptide sequences with distinct functions. Users can retrieve functional peptides specific to the species they are interested in. The length of peptides ranges from 5 to 200 amino acids. Each peptide in MFPPDB contains a maximum of three different therapeutic functions, but the number of peptides with three functions simultaneously is relatively small. Since, anti-bacterial peptides are extremely widespread in nature, as well as a large number of anti-bacterial peptides in the training samples, which resulted in a high number of anti-bacterial peptides in the predicted results (**Figure 2**). A total of 999,471 entries (67.4% of total) belongs to anti-bacterial peptides followed by anti-fungal peptide with 315,564 entries (21.3% of total identified peptides) (**Figure 2**). The number of peptides with dual functions, i.e., anti-bacterial and anti-fungal, is 73,373 entries (4.9% of total peptides identified). The database also includes 59,283 entries of anti-viral peptides, and 1.2% of peptides with anti-HIV and anti-viral functions. Additionally, we have also matched the predicted peptides identified in MFPPDB to the functional curated or structurally annotated therapeutic peptide datasets from nine state-of-the-art peptide databases currently accessible, including THPdb (Usmani et al., 2017), AHTPDB (Kumar et al., 2015), CPPsite 2.0 (Agrawal et al., 2016), APD3 (Wang et al., 2016), ACovPepDB (Zhang et al., 2022a), AntiTbPdb (Usmani et al., 2018), PlantPepDB (Das et al., 2020), YADAMP (Piotto et al., 2012) and SATPdb (Singh et al., 2016) (**Supplementary Table 1**). These databases totally comprise 46,319 peptides distributed in categories such as FDA-approved therapeutic peptides, anti-hypertensive peptides, cell-penetrating peptides, anti-microbial peptides, anti-coronavirus peptides, anti-tubercular peptides, plant-derived peptides (**Supplementary Table 1**). After matching roughly based on BLASTP (Altschul et al., 1990), we identified a total of 293,408 hits with 75,718 unique predicted plant peptides in MFPPDB against the peptides in the nine databases, indicating that those plant peptides possess the potential functions with the targeted terms in the database (**Supplementary Table 2**).

**Table 1:** Functional classification of the identified peptides and the dataset source used to train the model in MFPPDB.

Name	Full function	Count	References
ABP	Anti-bacterial	999471	(Xiao et al., 2021)
AFP	anti-fungal	315564	(Fang et al., 2019)
ABP, AFP	Anti-bacterial and anti-fungal	73373	(Xiao et al., 2021)
AVP	Anti-viral	59283	(Zhang and Zou, 2020)
AHIVP, AVP	Both anti-HIV and anti-viral	18526	(Xiao et al., 2021)
ABP, AVP	Both anti-bacterial and anti-viral	5412	(Xiao et al., 2021)
APP	Anti-parasitic	5068	(Zhang et al., 2022b)
APP, AVP	Both anti-parasitic and anti-viral	1750	(Xiao et al., 2021)
ABP, APP	Anti-bacterial and anti-parasitic	958	(Xiao et al., 2021)
AIP	Anti-inflammatory	920	(Khatun et al., 2020)
BBP	Blood-brain barrier	479	(Dai et al., 2021)
ABP, AHIVP, AVP	Anti-bacterial/-HIV/-viral	370	(Xiao et al., 2021)
AHIVP	Anti-HIV	341	(Xiao et al., 2021)
CPP	Cell-penetrating	298	(Zhang and Zou, 2020)
AAP	Anti-angiogenic	161	(Zhang and Zou, 2020)
ADP	Anti-diabetic	126	(Roy and Teron, 2019)
THP	Tumor-homing	55	(Shoombuatong et al., 2019)
ABP, AHIVP	Both anti-bacterial and anti-HIV	46	(Xiao et al., 2021)
AFP, AVP	Both anti-fungal and anti-viral	35	(Xiao et al., 2021)
SBP	Surface-binding	32	(Zhang and Zou, 2020)
AHP	Anti-hypertensive	25	(Xu et al., 2021)
ABP, AFP, APP	Anti-bacterial/-fungal/-parasitic	18	(Xiao et al., 2021)
ABP, APP, AVP	Anti-bacterial/-parasitic/-viral	17	(Xiao et al., 2021)
ATP	Anti-tubercular	17	(Jain et al., 2021)
DPPIP	Dipeptidyl peptidase IV	13	(Charoenkwan et al., 2020)
ADP, AIP	Anti-diabetic and anti-inflammatory	11	(Roy and Teron, 2019; Khatun et al., 2020)
QSP	Quorum-sensing	9	(Zhang and Zou, 2020)
ABP, ACP	Anti-bacterial and anti-cancer	6	(Xiao et al., 2021)
ACP	Anti-cancer	5	(Agrawal et al., 2021)
ACVP	Anti-coronavirus	3	(Pang et al., 2021)
AFP, APP	Anti-fungal and anti-parasitic	3	(Xiao et al., 2021)
ABP, AEP	Anti-bacterial/-endotoxin	2	(Xiao et al., 2021)
ABP, AFP, AVP	Anti-bacterial/-fungal/-viral	2	(Xiao et al., 2021)
ABP, ATP	Anti-bacterial and anti-tubercular	2	(Jain et al., 2021; Xiao et al., 2021)
AFP, BBP	Anti-fungal and blood-brain barrier	2	(Fang et al., 2019; Dai et al., 2021)
ABP, AIP	Anti-bacterial and anti-inflammatory	1	(Khatun et al., 2020; Xiao et al., 2021)
ABP, BBP	Anti-bacterial and blood-brain barrier	1	(Dai et al., 2021; Xiao et al., 2021)
ACP, AFP	Anti-cancer and anti-fungal	1	(Xiao et al., 2021)
ACVP, AVP	Anti-coronavirus and anti-viral	1	(Pang et al., 2021; Xiao et al., 2021)
AIP, CPP	Anti-inflammatory and cell-penetrating	1	(Khatun et al., 2020; Zhang and Zou, 2020)
BIP	Biofilm-inhibitory	1	(Fallah Atanaki et al., 2020)

We then carried out statistical analyses of the therapeutic peptide data stored in MFPPDB. We found that the top five plant species with most abundant therapeutic functions are *Triticum aestivum* (99,401 entries), *Hordeum vulgare* (58,941 entries), *Thinopyrum intermedium* (47,090 entries), *Zea mays* (33,732 entries), and *Camellia sinensis* (30,059 entries). There are more than 20 functional types of peptides were observed in 10 plant species, and only *Citrus clementina* contains 9 types of therapeutic functional peptides. About 68.5% of the plant species (83 out of total 121 species) contain 12 -16 types of therapeutic peptides. Among them, *Vitis vinifera* has the highest variety of therapeutic peptides with 26 therapeutic peptides. Both *Carica papaya* and *Triticum aestivum* have 24 types of therapeutic plant peptides. While, *Brachypodium mexicanum*, *Musa acuminata*, and *Oropetium thomaeum* have 21 types of functional peptides. *Cucumis sativus*, *Spinacia oleracea*, and *Trifolium pratense* contain 20 functional peptides. The statistics can be found in **Supplementary Figure 1**. MFPPDB provides researchers with a large variety of therapeutic plant peptides. If researchers want to find therapeutic peptides with multiple functional categories, they can focus on species such as *Vitis vinifera* and *Carica papaya*. If users want to obtain a large number of functional peptides in certain species, they can refer to *Triticum aestivum* and *Hordeum vulgare*. Depending on specific requirements, the development of functional peptides and peptide drugs can be supported in a targeted manner, thereby contributing to the application of peptide therapy in the medical field.

### 2.2 Implementation of MFPPDB

#### 2.2.1 Database construction

**Figure 3:**
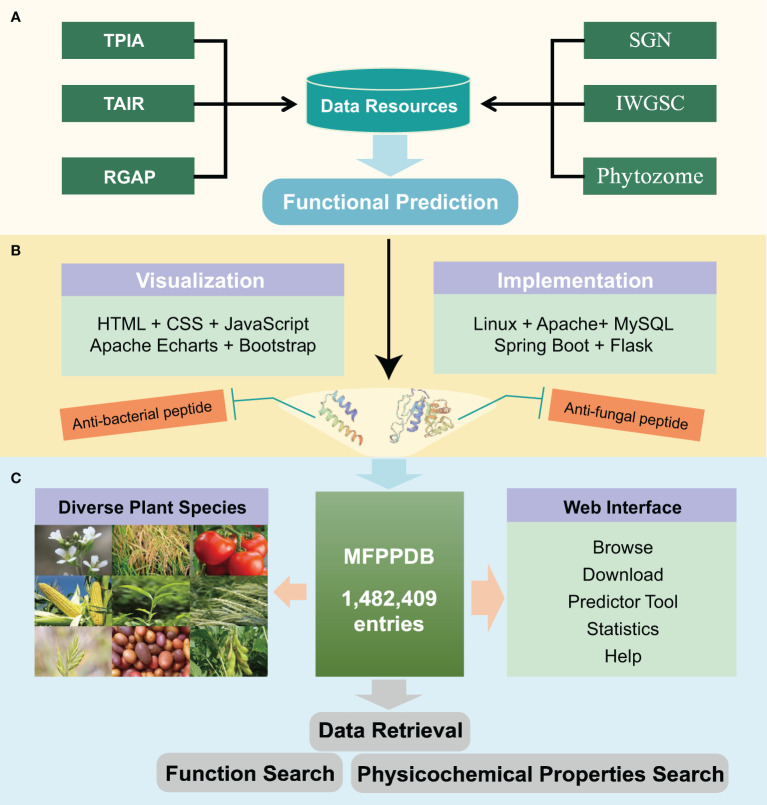
The overall architecture of the MFPPDB database. **(A)** The data resources of MFPPDB database, including public databases such as TPIA, TAIR, RGAP, SGN, IWGSC, and Phytozome. The formed data set is injected with plant peptides with single or multiple functions, such as anti-bacterial peptide, anti-fungal peptide, etc., into the database species through function prediction. **(B)** The tools used to build the website. The visualized MFPPDB database uses HTML, CSS, JavaScript, Apache ECharts and Bootstrap. The deployment environment and back-end implementation tools are Linux, Apache, MySQL, Spring Boot, and Flask. **(C)** The main interfaces (such as Browse, Download, Predictor, etc.) and retrieval tools (Function Search and Physicochemical Properties Search) contained in the MFPPDB database.

We developed the MFPPDB database using MySQL, Apache Tomcat web server, Java Spring Boot, and Python Flask tools. The environment required for the development and deployment of the application is integrated into the Tencent Cloud Linux server. Spring Boot is the main development tool for the web backend, while Python Flask is used to quickly deploy a multifunctional plant peptide predictor on the web server. All data constituting the MFPPDB database are stored on the relational database management system MySQL server (version 8.0.31). HTML, CSS, and JavaScript are used for developing web front-end pages, with Bootstrap as the front-end framework. Apache Echarts is used for data visualization, which presents rich multifunctional plant peptide data to users. The architecture of MFPPDB is shown in **Figure 3**.

#### 2.2.2 Search module

**Figure 4:**
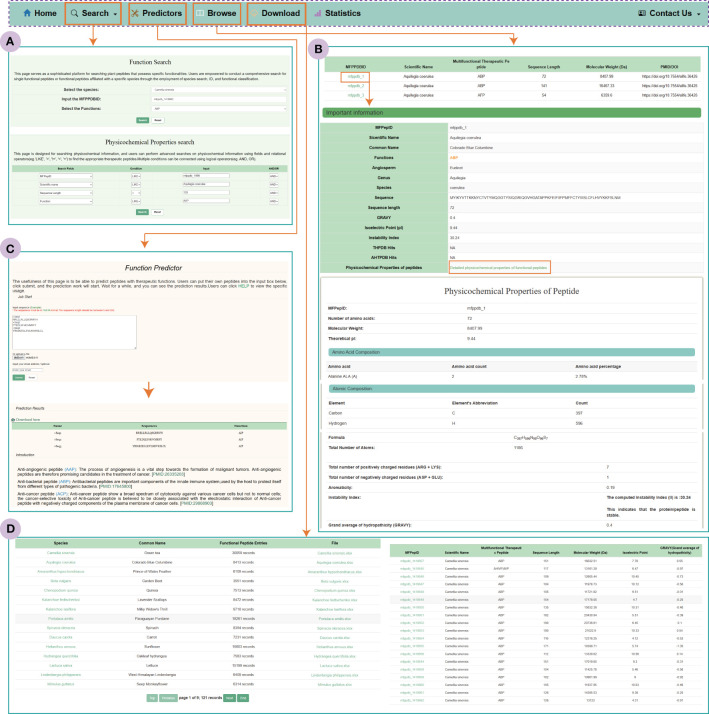
The MFPPDB database interfaces. **(A)** Search tools include “Function Search” and “Physicochemical Properties Search”. **(B)** “Predictor” interface and prediction result page. **(C)** The “Browse” interface. The figure shows the important information and Physicochemical information contained in the functional peptide taking ‘mfppdb_1’ as an example. **(D)** Download page for Functional peptide data.

The search module in MFPPDB provides users with two search modes: functional search and physicochemical property search (**Figure 4**). With the ‘Functional Search’ module, users can search against each individual plant species for peptides with specific class of functions, e.g., ‘ABP’ or ‘ABP, AHIVP’ in the original predicted datasets or the functional matched datasets. By selecting the species name, MFPPDB ID or the functions in function search, users can easily retrieve the detail information of target functional peptides. On the other hand, the ‘Physicochemical Properties Search’ module allows users to perform complex queries and use conditional operators on different fields, as well as submit queries using Boolean expressions (such as “AND” or “OR”). The fields in this module contain essential functional and physicochemical properties of therapeutic peptides, such as function, isoelectric point, sequence length, instability index, and so on. By utilizing these conditions simultaneously, users can easily find the therapeutic peptides of their interest. To help users obtain the possible functions of the total predicted plant peptides, we also integrated the information of the matched peptides in MFPPDB to other nine databases into the search module, in which users can easily search and retrieve the matched peptides. The output is an additional column in the search page that shows the functional entries in the selected databases.

#### 2.2.3 Browse module

The Browse page is the primary way for users to explore the MFPPDB database and discover peptides with various functional characteristics. Upon accessing this page, the users will be presented with a table containing peptide’s ID, scientific names, molecular weights, functions, and corresponding literature references (PMID and DOI). By clicking on a MFPPDB ID, users can access the detail information of the functional peptide, includes the therapeutic function, peptide sequence, sequence length, isoelectric point, and the description of the peptides matched to functional curated peptides in other nine peptide databases. Additionally, an interface is available for users to access more comprehensive physicochemical properties information for each peptide.

### 2.3 Predictor module

We have also incorporated a user-friendly prediction tool into the web server, which predicts the candidate function of plant peptides. The prediction server can effectively predict functional characteristics of plant peptides in both single and batch mode. Users can input the plant peptide sequence in FASTA format into the provided input box or submit the FASTA file containing the peptide sequence, and then click the submit button to initiate the prediction process. The prediction process only takes a few minutes, and the predicted results are presented to the user in the form of a table on the result page. Users can easily download the predicted results or receive them via email. The prediction algorithm used by the tool is based on the novel multi-label predictor, PrMFTP, which uses deep neural networks and a multi-head self-attention mechanism (Yan et al., 2022). The prediction tool in MFPPDB has been retrained using therapeutic peptides with sequence length between 5 and 200 amino acids following the original algorithm from PrMFTP, ensured the accuracy and reliability.

### 2.4 Download module

As a free public functional peptide database, the MFPPDB provides users with a page for downloading all the data used in the database. The Download page is displayed to the user in the form of a table, where they can observe the common name, number of peptides, and download interface corresponding to each species. MFPPDB provides data to users in the Excel format, which contains a lot of information about the current species, such as MFPPDBID, peptide sequence, sequence length, functional characteristics, scientific name, etc. We also provide an interface on this page that allows quick access to the functional peptide information of the current species. Users can simply click on the species to browse the peptide information for the species they interest.

## 3 Limitations and future development

The MFPPDB database is useful for the large number of plant peptides predicted to have multiple therapeutic functions. After the prediction, we also tried to match the possible functional descriptions of those predicted plant peptides to the functional verified or curated peptides in other nine state-of-the-art peptide databases. Indeed, we found at least 20% of the predicted plant peptides show the potential functions as with the hits in the nine databases. However, it should be noted that the matching is still based on peptide sequence alignments, even the matching targets were the experimentally validated or curated peptides. It owns to those excellent developed functional curated databases containing therapeutic peptides, such as THPdb, AHTPDB, CPPsite 2.0, APD3, ACovPepDB, AntiTbPdb, PlantPepDB, YADAMP and SATPdb. These results indicated that the plant peptides in MFPPDB have therapeutic functions in potential despite of the fact that the functional validation need to be experimentally conducted in the future. Among these databases, the PlantPepDB serves a similar function with MFPPDB, in which both databases focus on plant peptides having different functions and therapeutic activities. Compared to the abundant predicted or verified plant peptides in MFPPDB, although PlantPepDB only contains 3,848 peptide entries; however, the peptides in PlantPepDB are manually curated from 11 databases and 835 published research articles. The functions of these peptides might be more detail and accurate than those in MFPPDB. After matching and verifying the predicted peptides with those known manually curated plant peptides in PlantPepDB and other databases, we believe that these predicted and verified plant peptides in MFPPDB are also the important sources for therapeutic usage.

In the current version of MFPPDB, we have included a total of 1,482,409 peptides, in which 293,408 were potentially verified with therapeutic functions collected from the representative plant species in nature. In the further version of MFPPDB, we plan to expand our collection to include more categories of plants, such as bryophytes, ferns, gymnosperms, etc. We also aim to obtain more properties of the peptide sequences, such as their secondary and tertiary structures, and to add other effective computational tools to the MFPPDB. More importantly, we will also keep trying to verify or validate the functions of the predicted plant peptides either computationally or experimentally, thus providing more comprehensive and accurate information to assist biologists in their scientific research.

## 4 Materials and methods

### 4.1 Peptide sequence resources

The multifunctional plant peptide database consists of plants from monocots and dicots of 121 species across 98 genera of angiosperms. The database includes peptide sequences from model plants such as *Arabidopsis* (from the TAIR database), rice (from the Rice Genome Annotation Project), *Solanum lycopersicum* (from the *Solanaceae* Genomics Network), *Solanum tuberosum* (from SpudDB), *Triticum aestivum* (from the International Wheat Genome Sequencing Consortium), and *Camellia sinensis* (from the TPIA database). The peptide sequences of the other 115 angiosperms were obtained from Phytozome (https://phytozome-next.jgi.doe.gov/).

### 4.2 Curation and compilation of plant peptides

After obtaining the original plant peptide sequences from multiple public databases, we performed preprocessing to eliminate non-compliant peptide sequences. Firstly, we discarded plant peptides containing non-standard amino acids in their sequences. Secondly, we removed plant peptide sequences containing repetitive amino acids. Lastly, we extracted plant peptides with sequence lengths between 5 and 200 amino acids. Subsequently, the peptide sequences were input into the function prediction model to identify plant peptides with multiple therapeutic functions, which were then included in the MFPPDB database as significant data. Matching of the predicted plant peptides from MFPPDB to other curated datasets from nine public databases were conducted using BLASTP (Altschul et al., 1990) with E-value less than 1E-3 and identity greater than 50%.

### 4.3 Physicochemical properties of plant peptides

All the identified peptides in the MFPPDB library were carefully analyzed using computational tools to calculate the physicochemical properties of each functional peptide from the peptide sequence input. The atomic composition of peptides includes carbon, hydrogen, oxygen, nitrogen, and sulfur were calculated using the Proteomics Toolkit (http://db.systemsbiology.net/proteomicsToolkit/FragIonServlet.html). We obtained the source code on Github and ran it in the Java interpretation environment. The results of the operation output the atomic composition information of the peptide in the form of a table. We also used the “BioSeqUtils” module provided by Biopython (Cock et al., 2009) and imported the ProtParam program to analyze various attributes of plant peptide sequences with multiple therapeutic functions, such as the number of amino acids, amino acid percentages, isoelectric points, molecular weight, hydrophilicity (GRAVY) index, instability index, aromaticity, secondary structure fraction, and atomic composition. The number of positively and negatively charged residues was calculated based on the previously calculated amino acid counts.

## 5 Conclusions

In current report, we establishment the MFPPDB database, enables the extraction of therapeutic peptide information scattered across various plant species using a novel multi-functional therapeutic peptides prediction tool— PrMFTP. Based on those well predicted and pre-functional verified peptides, we integrate it into a comprehensive resource library and presenting the detail information or characteristics of the peptides to researchers in an intuitive way on current MFPPDB version. It greatly reduces the time and energy wasted in collecting data for bioinformatics researchers when exploring plant peptides or proteins of a certain species, allowing them to focus more on verifying the function of peptides from current database and by additional experimental methods. We believe that the development of MFPPDB will accelerate research on plant peptides with therapeutic functions.

## Data availability statement

The original contributions presented in the study are included in the article/**Supplementary Material**. Further inquiries can be directed to the corresponding authors.

## Author contributions

KL and WT conceived the study and supervised all parts of the project. YY and HW collected samples and sequence data. YY, HW, and YG analyzed the data and constructed the database. YY, KL, and WT wrote and revised the manuscript. All authors contributed to the article and approved the submitted version.

## Supplementary material

SUPPLEMENTARY FIGURE 1 The number of functional peptides contained in each species.
