# Supplementary figures and images for: MFPPDB: a comprehensive multi-functional plant peptide database
Source: Front Plant Sci. 2023 Oct 16;14:1224394. doi: 10.3389/fpls.2023.1224394 (PMC10613858; doi:10.3389/fpls.2023.1224394)

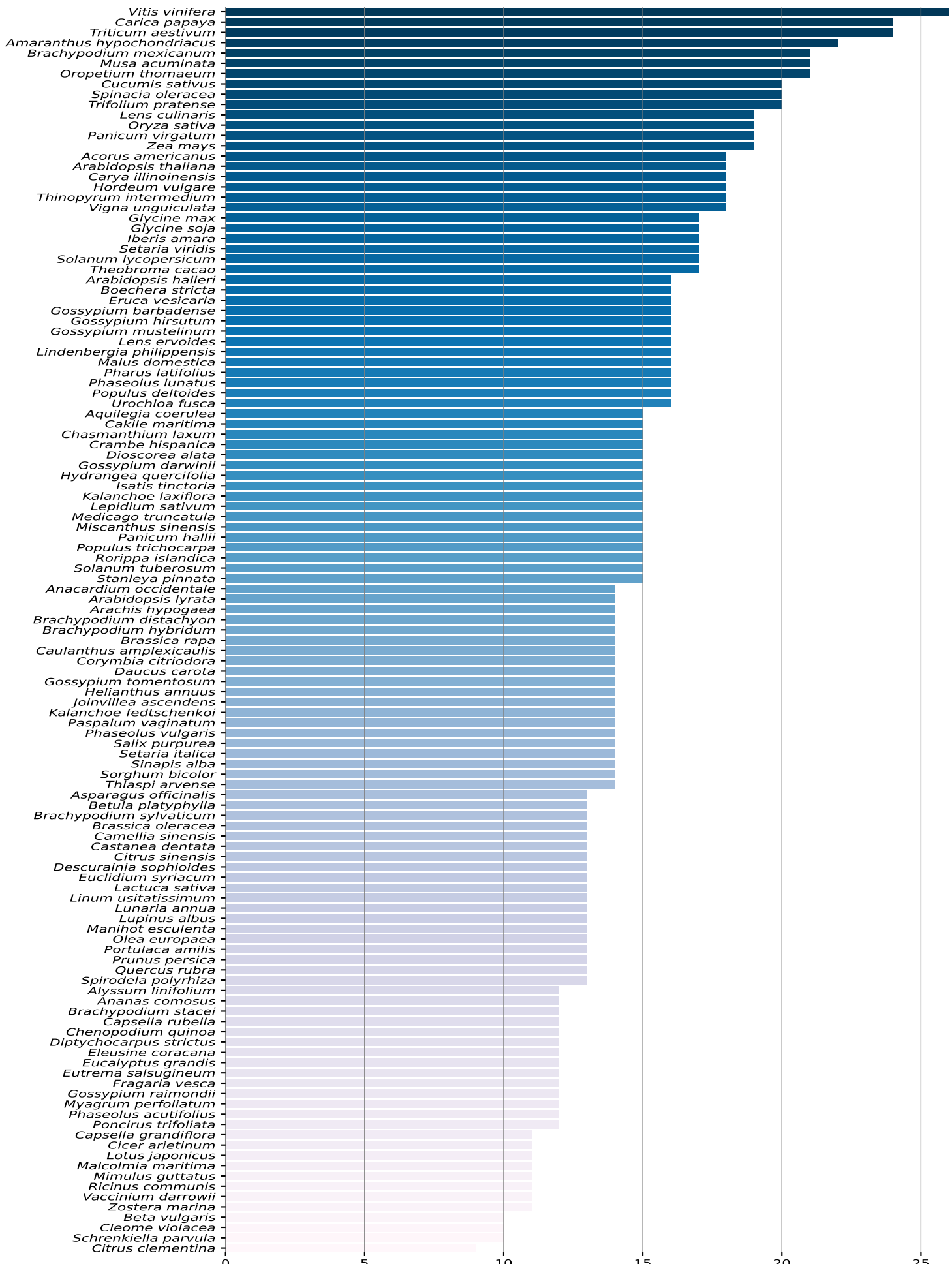

Numbers

Supplement: SUPPLEMENTARY FIGURE 1 — The number of functional peptides contained in each species. [file DataSheet_1.pdf]
